# Ticks and Tick-Borne Pathogens of the Caribbean: Current Understanding and Future Directions for More Comprehensive Surveillance

**DOI:** 10.3389/fcimb.2017.00490

**Published:** 2017-11-29

**Authors:** Mathilde Gondard, Alejandro Cabezas-Cruz, Roxanne A. Charles, Muriel Vayssier-Taussat, Emmanuel Albina, Sara Moutailler

**Affiliations:** ^1^UMR BIPAR, Animal Health Laboratory, ANSES, INRA, Ecole Nationale Vétérinaire d'Alfort, Université Paris-Est, Maisons-Alfort, France; ^2^CIRAD, UMR ASTRE, Petit-Bourg, France; ^3^Faculty of Science, University of South Bohemia, Ceské Budejovice, Czechia; ^4^Biology Center, Institute of Parasitology, Czech Academy of Sciences, Ceské Budejovice, Czechia; ^5^Faculty of Medical Sciences, School of Veterinary Medicine, University of the West Indies, Mt. Hope, Trinidad and Tobago; ^6^INRA, UMR 1319 ASTRE, Montpellier, France

**Keywords:** tick-borne pathogens, ticks, Caribbean, epidemiology, new high-throughput technologies, surveillance

## Abstract

Ticks are obligate hematophagous arthropods of significant importance to human and veterinary medicine. They transmit a vast array of pathogens, including bacteria, viruses, protozoa, and helminths. Most epidemiological data on ticks and tick-borne pathogens (TBPs) in the West Indies are limited to common livestock pathogens such as *Ehrlichia ruminantium, Babesia* spp. (i.e., *B. bovis* and *B. bigemina*), and *Anaplasma marginale*, and less information is available on companion animal pathogens. Of note, human tick-borne diseases (TBDs) remain almost completely uncharacterized in the West Indies. Information on TBP presence in wildlife is also missing. Herein, we provide a comprehensive review of the ticks and TBPs affecting human and animal health in the Caribbean, and introduce the challenges associated with understanding TBD epidemiology and implementing successful TBD management in this region. In particular, we stress the need for innovative and versatile surveillance tools using high-throughput pathogen detection (e.g., high-throughput real-time microfluidic PCR). The use of such tools in large epidemiological surveys will likely improve TBD prevention and control programs in the Caribbean.

## Introduction

Ticks are obligate hematophagous arthropods of significant econonomic and sanitary importance affecting human and animal health worldwide. While acquiring a blood meal, ticks can both directly and indirectly harm the host. They are known to induce severe toxic conditions such as paralysis, allergies, abscesses, anemia, immunosuppression, and skin deterioration at the bite site (Mans et al., 2004). More importantly, ticks can also transmit severe infections as they can be vector of various pathogens (De la Fuente et al., 2017). Ticks are divided into three families: the Nuttalliellidae (comprising a single genus and species), the Ixodidae or hard ticks (that includes 14 genera and ~700 species), and the Argasidae or soft ticks (that includes 5 genera and ~200 species) (Guglielmone et al., 2010; Manzano-Román et al., 2012). Nearly 10% of the ~900 known tick species can transmit pathogens, and amongst all arthropods, ticks transmit the greatest variety of pathogenic microorganisms, including bacteria, viruses, protozoa, and helminths (Jongejan and Uilenberg, 2004).

Despite the high burden of animal and zoonotic infectious diseases in the West Indies, implementing integrative approaches to manage and control ticks and their pathogens remains challenging. Insufficient epidemiological knowledge on tick diversity and the tick-borne pathogens (TBPs) circulating in the region, combined with a lack of expertise in TBD management, limits the development of effective control strategies (Pegram et al., 2007). Moreover, the Caribbean region is a cosmopolitan area, at the crossroads of intercontinental exchanges between the Americas, Europe, and Africa, which thus poses risks of tick and TBP introduction and dispersal, especially through animal movement (legal or illegal trade and bird migration) (George et al., 2002). Therefore, new insights into tick and TBP epidemiology are needed to address TBD emergence in this area. Indeed, accurate knowledge about the diversity of TBPs circulating in a specific region is a critical step toward implementing effective TBD prevention and control programs.

Following a comprehensive review of the reported tick species and TBPs of medical and veterinary importance in the Caribbean, we discuss the challenges of TBD surveillance and management in the Caribbean. In addition, we highlight the importance of applying novel technologies for multiple parallel pathogen detection to support tick and TBP control programs.

## Tick vectors in the Caribbean

The Caribbean tick fauna is composed of both endemic species, and exotic species that have mostly been introduced by animal movements. Some tick species have been naturally imported by migratory or non-migratory birds originating from North, Central, or South America, while others were introduced by humans during the colonization of the Americas with the arrival of infested cattle and dogs from Europe, Africa, and Asia (Morel, 1966; De la Cruz, 2001). Thus far, 56 tick species have been recorded in the Caribbean. They belong to 10 genera, and two famillies (Argasidae and Ixodidae) including 15 species of *Ornithodoros*, 10 species of *Antricola*, 17 species of *Amblyomma*, 3 species each of *Argas, Ixodes*, and *Rhipicephalus*, 2 species of *Haemaphysalis*, and 1 species each of *Parantricola, Dermacentor (Anocentor)*, and *Aponomma* (De la Cruz, 2001; Basu and Charles, 2017). The life cycle for both male and female ticks includes three developmental stages (excluding eggs): larvae, nymphs (one nymphal stage in Ixodidae and several nymphal stages in Argasidae), and adults. Each of these stages can potentially acquire and transmit pathogens while feeding on a host. According to the tick species and to the pathogen, there are several ways of pathogen transmission from one tick to another and from one tick to a new host. Some tick species are able to maintain horizontally pathogens within the same generation (1) Tick may be able to transmit transstadially (or interstadially) pathogens, meaning that one tick developmental stage can acquire and then transmit pathogens to the next developmental stage, maintaining pathogens through the molts (2) Tick may be able to intrastadially transmit pathogens to the host, meaning that tick may be able to transfer pathogens from one host to another during the same developmental stage (3) Tick may be able of venereal transmission, when male tick transmits pathogens to the female tick during the mating (Connell and Hall, 1972; Parola and Raoult, 2001; Bremer et al., 2005; Ueti et al., 2008). Some tick species are also able of vertical maintenance, transmitting pathogens to the next generation. Infected female ticks may be able to transmit pathogens transovarially to their offspring, leading to infected larvae (Parola and Raoult, 2001). Finally, tick may be able of non-systemic transmission. When feeding on a host, ticks tend to form clusters (known as co-feeding), a behavior that—apart from facilitating blood feeding and mating—also assists in pathogen transmission from an infected tick to an uninfected one sharing the same blood meal (Randolph et al., 1996). During co-feeding, ticks can transmit bacterial and viral pathogens to each other without the host bacteremia or viremia (Randolph et al., 1996; Belli et al., 2017). Finally, ticks can transmit biologically and mechanically pathogens to a new host. The majority of TBPs are transmitted to the host through the tick salivary glands via the saliva. Non-salivary TBP transmission can also occur by contamination of the feeding site with infectious regurgitated midgut contents, feces, coxal fluid, or contaminated mouthparts (Parola and Raoult, 2001). Vertebrate host infection can also occur by orally ingesting infected ticks, as demonstrated for *Hepatozoon* spp. sporozoites transmission (Mathew et al., 1998; Baneth et al., 2001; Modrý et al., 2017).

Owing to their health impact, the most studied tick species in the West Indies are those associated with TBP transmission to livestock or pets. However, even though tick tropism for certain hosts is well-documented; these same ticks are able to parasitize different host species, including humans, thus posing a zoonotic risk (Parola et al., 1999; Dantas-Torres, 2010). Other tick species—mostly occurring in the wildlife population—are present within the Carribean, but have not received much interest so far (De la Cruz, 2001; Basu and Charles, 2017). As the majority of emerging diseases originate from wildlife reservoirs, characterizing the diversity and ecology of ticks present in such wild environments should be addressed (Dantas-Torres et al., 2012; Baneth, 2014). The tick species described in this review are significantly involved in the epidemiology of animal TBDs (Table 1).

**Table 1:** Tick-borne pathogens and suspected tick vectors reported within the Caribbean.

**Pathogens**	**Caribbean distribution**	**Infection (TBDs)**	Vector^*^	**Reported host**	**Detection method**	**References**
*Anaplasma marginale*	Antigua, Barbados, Cuba, Dominica, Dominican Republic, Grenada, Guadeloupe, Haiti, Jamaica, Martinique, Montserrat, Puerto Rico, St Kitts and Nevis, St Lucia, St Martin, St Vincent, Trinidad	Anaplasmosis	*Rh. microplus*	Cattle	Serology	Camus and Montenegro-James, 1994; Camus and Barre, 1995
*Anaplasma phagocytophilum*	Puerto Rico	Anaplasmosis (Granulocytic Anaplasmosis)	*Ixodes* spp	Dogs	Serology	McCown et al., 2013
*Anaplasma platys*	Cuba, Grenada, Haiti, St Kitts, Trinidad,	Anaplasmosis (Canine Cyclic Thrombocytopenia)	*Rh. sanguineus, A.cajennense*	Dogs	Serology/Molecular biology	Georges et al., 2008; Yabsley et al., 2008; Kelly et al., 2013; Loftis et al., 2013; Silva et al., 2016; Starkey et al., 2016
*Bartonella vinsonii* subspp. *berkhoffii*	Grenada, Martinique	Canine and human endocarditis	*Rh.sanguineus*	Dogs	Serology/Molecular biology	Boulouis et al., 2005; Yabsley et al., 2008
*Borrelia burgdorferi* sensu lato	Cuba	Borreliosis (Lyme disease)	*A.cajennense*	Humans	Serology	Rodríguez et al., 2004, 2012
*Borrelia Relapsing Fever group (Borrelia hermsii)*	US Virgin Islands	Borreliosis (Relapsing fever)	*Ornithodoros* spp	Humans	Serology	Flanigan et al., 1991
*Coxiella burnetii*	Puerto Rico	Q fever	*D. nitens*	Cattle	ND	Tamsitt and Valdivieso, 1970
*Ehrlichia canis*	Aruba, British West Indies, Grenada, Haiti, Puerto Rico, St Kitts and Nevis, Trinidad, Turks and Caicos Islands	Ehrlichiosis (Canine Monocytotic Ehrlichiosis)	*Rh. Sanguineus*	Canids, Cats	Serology/Molecular biology	Morel, 1967; Georges et al., 2008; Hoff et al., 2008; Yabsley et al., 2008; Loftis et al., 2013; McCown et al., 2013; Starkey et al., 2016
*Ehrlichia canis* or closely related species	Dominica, Grenada, Montserrat, St Kitts and Nevis	Ehrlichiosis	ND	Cattle, Sheep, Goats	Molecular biology	Zhang et al., 2015a
*Ehrlichia ruminatium*	Antigua, Guadeloupe, Marie Gualante	Ehrlichiosis (Heartwater, Cowdriosis)	*A. variegatum*	Cattle	Serology	Camus and Barre, 1995; Vachiéry et al., 2008; Kelly et al., 2011
*Panola Mountain Ehrlichia* sp.	Dominica, St Kitts	Ehrlichiosis	*A. variegatum*	Domestic animals	Molecular biology	Loftis et al., 2016
*Candidatus* Mycoplasma haematoparvum	Trinidad	Hemotropic mycoplasmosis	*Rh.sanguineus*	Dogs	Molecular biology	Barker et al., 2010
*Mycoplasma haemocanis*	Trinidad	Hemotropic mycoplasmosis	*Rh. sanguineus*	Dogs	Molecular biology	Barker et al., 2010
*Mycoplasma haemofelis*	Trinidad	Hemotropic mycoplasmosis	*Rh. sanguineus*	Cats	Molecular biology	Georges et al., 2008
*Candidatus* Mycoplasma haemominutum	Trinidad	Hemotropic mycoplasmosis	*Rh. sanguineus*	Cats	Molecular biology	Georges et al., 2008
*Mycoplasma wenyonii*	Cuba	Hemotropic mycoplasmosis	ND	Cattle	ND	Rodríguez et al., 1989a
*Mycoplasma ovis*	Cuba	Hemotropic mycoplasmosis	ND	Sheep	Serology	Rodríguez et al., 1989b
*Rickettsia africae*	Antigua, Dominica, Guadeloupe, Martinique, Montserrat, St Kitts and Nevis, St Lucia, U.S. Virgin Islands	Rickettsiosis (African tick bite fever)	*A.variegatum*	Humans, Cattle, Goats, Sheep	Serology/Molecular biology	Parola et al., 1998, 1999; Parola and Barre, 2004; Robinson et al., 2009; Kelly et al., 2010a
*Rickettsia conorii*	Guadeloupe	Rickettsiosis (Mediterranean spotted fever)	*A. variegatum*	Humans	Serology	Morel, 1967
*Rickettsia felis*	Dominica, St Kitts	Rickettsiosis	*Ctenocephalides felis*	Cats	Molecular biology	Kelly et al., 2010b
*Rickettsia typhi*	Puerto Rico	Rickettsiosis (Murine typhus)	*Xenopsylla cheopis*	Rodents	ND	Tamsitt and Valdivieso, 1970
*Babesia bigemina*	Antigua, Barbados, Cuba, Dominica, Dominican Republic, Grenada, Guadeloupe, Haiti, Jamaica, Martinique, Montserrat, Puerto Rico, St Kitts and Nevis, St Lucia, St Martin, St Vincent, Trinidad	Babesiosis	*Rh. microplus*	Cattle	Serology/Molecular biology	Camus and Montenegro-James, 1994; Camus and Barre, 1995; Li et al., 2015
*Babesia bovis*	Antigua, Barbados, Cuba, Dominica, Dominican Republic, Grenada, Guadeloupe, Haiti, Jamaica, Martinique, Montserrat, Puerto Rico, St Kitts and Nevis, St Lucia, St Martin, St Vincent, Trinidad	Babesiosis	*Rh. microplus*	Cattle	Serology/Molecular biology	Camus and Montenegro-James, 1994; Camus and Barre, 1995; Li et al., 2015
*Babesia caballi*	Grenada, Guadeloupe, Martinique, Montserrat, St Kitts and Nevis, Trinidad	Piroplasmosis	*D. nitens*	Equids, Goats, Sheep	Serology/Molecular biology	Morel, 1967; Asgarali et al., 2007; Li et al., 2015;
*Babesia (canis) rossi*	Montserrat	Babesiosis	*Rh.sanguineus, Rh. turanicus*	Goats	Molecular biology	Li et al., 2015
*Babesia (canis) vogeli*	Dominica, Grenada, Haiti, Montserrat, St Kitts and Nevis, Trinidad	Babesiosis	*Rh. sanguineus, Rh. turanicus*	Dogs, Cats, Cattle, Sheep, Goats	Serology/Molecular biology	Georges et al., 2008; Yabsley et al., 2008; Kelly et al., 2013; Li et al., 2015; Starkey et al., 2016;
*Babesia gibsoni*	Dominica, St kitts	Babesiosis	*Rh.sanguineus, Rh.turanicus*	Dogs, Cattle, Sheep, Goat, Equids	Molecular biology	Kelly et al., 2013; Li et al., 2015
*Babesia vulpes*	Montserrat	Babesiosis	ND	Goats, Sheep	Molecular biology	Zhang et al., 2015b
*Hepatozoon canis*	Aruba, Grenada, Haiti, St Kitts, Trinidad	Hepatozoonosis	*Rh. sanguineus*	Dogs	Serology/Molecular biology	Yabsley et al., 2008; Kelly et al., 2013; Starkey et al., 2016; Sant et al., 2017
*Theileria equi*	Dominica, St kitts and Nevis, Trinidad	Piroplasmosis	*D. nitens*	Equids, Cattle, Sheep, Goats	Serology/Molecular biology	Asgarali et al., 2007; Zhang et al., 2015b
*Theileria mutans*	Cuba, Guadeloupe, Martinique	Theileriosis	*A. variegatum*	Cattle	Serology (IFA)	Uilenberg et al., 1983; Rodríguez et al., 1989b; Alonso et al., 1992
*Theileria parva*	Guadeloupe	Theileriosis	*A. variegatum*	Cattle	ND	Morel, 1967
*Theileria sp. B15a*	Grenada	Theileriosis	ND	Cattle	Molecular biology	Zhang et al., 2015b
*Theileria sp. NG-2013a*	Nevis	Theileriosis	ND	Goats	Molecular biology	Zhang et al., 2015b
*Theileria sp. OT3*	Montserrat	Theileriosis	ND	Sheep, Goats	Molecular biology	Zhang et al., 2015b
*Theileria sp. YW-2014*	St Kitts	Theileriosis	ND	Equids	Molecular biology	Zhang et al., 2015b
*Theileri velifera*	Guadeloupe	Theileriosis	*A. variegatum*	Cattle	Serology	Uilenberg et al., 1983
African swine fever	Cuba, Dominican Republic, Haiti	African swine fever	*Ornithodoros* spp	Swine	ND	Butler and Gibbs, 1984; Penrith, 2009
Estero Real	Cuba	ND	*C. tadaridae*	ND	Isolation	Málkov et al., 1985
Hugues Virus	Cuba, Trinidad	ND	*C. denmarki*	Seabirds	Isolation	Aitken et al., 1968; Danielová et al., 1982
Soldado Virus	Trinidad	ND	*Carios* spp	Seabirds	Isolation	Jonkers et al., 1973
Wad Medani Virus	Jamaica	ND	*A.cajennense*	ND	Isolation	Belle et al., 1980

* *Suspected or characterized tick vector*.

### Ixodid tick vectors in the Caribbean

Ixodidae represent approximately 80% of the world's tick fauna. They can be morphologically distinguished from soft ticks due to the presence of a sclerotized scutum, and a capitulum harboring the mouthparts originating from the anterior face (Estrada-Peña, 2015). Ixodidae may be one-, two-, or three-host species depending on the number of host animals they need to complete their life cycle (Jongejan and Uilenberg, 2004). Hard ticks can present endophilous behavior, living in proximity with the host in a protected environment, or/and exophilous behavior, where they live in an exposed environment, questing for a host (Estrada-Peña, 2015). Among the ixodid ticks implicated in Caribbean TBDs, the *Rhipicephalus* spp. *Amblyomma* spp., and *Dermacentor* spp. are the most commonly found (Table 1).

*Rhipicephalus (Boophilus) microplus*, the tropical cattle tick, is considered to be the most important parasitic livestock tick worldwide. Although mainly associated to cattle, *Rh. microplus* is able to feed on a variety of hosts including buffaloes, horses, donkeys, goats, sheep, deer, pigs, dogs, and some wild animals (Walker et al., 2003; Ghosh et al., 2007). Several cases of human paratization by *Rh. microplus* have been reported, but it is uncommon (Guglielmone et al., 2006; Lamattina and Nava, 2016). *Rh. microplus* are one-host ticks, meaning that they do not need to fall off of a host to molt, and specimens can spend their entire life cycle on the same host. They are well distributed within the Caribbean, reported in both the Greater and Lesser Antilles (Camus and Barre, 1995). The cattle tick is mainly implicated in the transmission of bacteria and protozoa such as *Anaplasma marginale, Babesia* spp., and *Theileria* spp. A national survey in 2000 reported that bovine babesiosis associated with *Rh. microplus* infestations caused economic losses of US $6.7 million in the Puerto Rican cattle industry (Urdaz-Rodríguez et al., 2009). Two successive tick eradication programs have been conducted in Puerto Rico, however due to many technical and socio-economic limitations, both projects failed and were abandoned (Crom, 1992; Pegram et al., 2000).

*Amblyomma variegatum*, the tropical bont tick, is a three-host tick widely distributed throughout the Caribbean, parasitizing various vertebrate hosts which are mainly ruminants such as cattle and goats, and represents a major constraint to animal farming (Barré and Garris, 1990). They create deep skin lesions via their long mouthparts, and thus favor the development of secondary infections such as acute dermatophilosis. *Amblyomma variegatum* is the vector of pathogenic bacteria such as *Ehrlichia* spp. and *Rickettsia* spp.; and protozoa such as *Theileria* spp. (Uilenberg et al., 1983; Camus and Barré, 1992; Parola et al., 1999; Loftis et al., 2016). In 1986, the economic importance of *A. variegatum* and associated animal diseases in the Lesser Antilles was estimated to be US $4.6 million per year (Camus and Barre, 1995). The tropical bont tick is still widespread within the Caribbean despite eradication attempts conducted in the English Lesser Antilles (Pegram et al., 2004).

*Amblyomma cajennense*, the Cayenne tick, is a three-host tick parasitizing a wide range of hosts, including humans (Estrada-Peña et al., 2004). Widely distributed throughout the Neotropical area of the Americas, *A. cajennense* is the second-most economically important tick species in South America (Camus and Barré, 1995). However, within the Caribbean, the distribution of *A. cajennense* seems to be restricted to Cuba, Jamaica, and Trinidad, where they are suspected to transmit *Ehrlichia* spp., *Rickettsia* spp., and equine piroplasms (Estrada-Peña et al., 2004; Scoles and Ueti, 2013).

*Rhipicephalus annulatus* shares *Rh. microplus'* ability to transmit both *Babesia* spp. and *Anaplasma* spp. This tick can be mistaken for *Rh. microplus* due to their morphological similarities. Although some studies reported the presence of *Rh. annulatus* in the Caribbean, its prevalence is not well-characterized, nor its potential role as a TBD vector in the area (Morel, 1966; De la Cruz, 2001; Basu and Charles, 2017).

*Rhipicephalus sanguineus*, the brown dog tick, is a three-host tick widely distributed within the Caribbean and which commonly parasitizes dogs (Morel, 1966; De la Cruz, 2001; Basu and Charles, 2017). *Rh. sanguineus* is an endophilic (adapted to indoor living) tick species, presenting threats to both human and animal health due to its ability to transmit *Ehrlichia, Anaplasma, Rickettsia*, and *Babesia* species (Dantas-Torres, 2010).

*Dermacentor (Anocentor) nitens*, the tropical horse tick, is a one-host tick that mainly parasitizes equines (Rodrigues et al., 2017). *D. nitens* is suspected to be the vector of *Babesia caballi* and *Theileria equi*, the two causal agents of equine piroplasmosis (Asgarali et al., 2007; Li et al., 2015; Zhang et al., 2015b).

### Argasid tick vectors in the Caribbean

Argasidae family taxonomy is still controversial due to extreme diversity and the absence of consensus guidelines for morphological identification. However, soft ticks do present numerous biological and ecological features that easily distinguish them from the Ixodidae. Argasid ticks do not possess a scutum, but a leathery integument, and their capitulum with mouthparts originating from the ventral face (Estrada-Peña, 2015). Generally, the parasitic stages feed on the host for several times over a short period (minutes to hours), ingesting a relatively small amount of blood per meal, and then go back to their nest (Jongejan and Uilenberg, 2004). It was thought that soft ticks have high host specificity, however, it has now been suggested that most soft ticks actually demonstrate indiscriminate host feeding. Such apparent variation in host preference probably reflects microhabitat preference and host availability within each microhabitat (Vial, 2009). Indeed Argasidae are typically defined by their endophilous behavior. Some species are endophilous nidicoles, adapted to living in their microhabitats by colonizing the nests and burrows of their hosts, and others are geophilous nidicoles, preferring locations near host habitations (Vial, 2009). Soft ticks mainly harbor tick-borne viruses, such as Hughes and Soldado virus, and some bacteria, such as *Borrelia* spp. responsible for human and animal disease (Labuda and Nuttall, 2004; Manzano-Román et al., 2012). Reports of African swine fever virus during outbreaks in Cuba and Hispaniola (Haiti and Dominican Republic) (Table 1) were suspected to be transmitted by *Ornithodoros* spp. (Penrith, 2009). However, data on the diversity of soft ticks and associated pathogens in the Caribbean are scarce.

## TBPs and TBDs reported within the Caribbean

**Figure 1:**
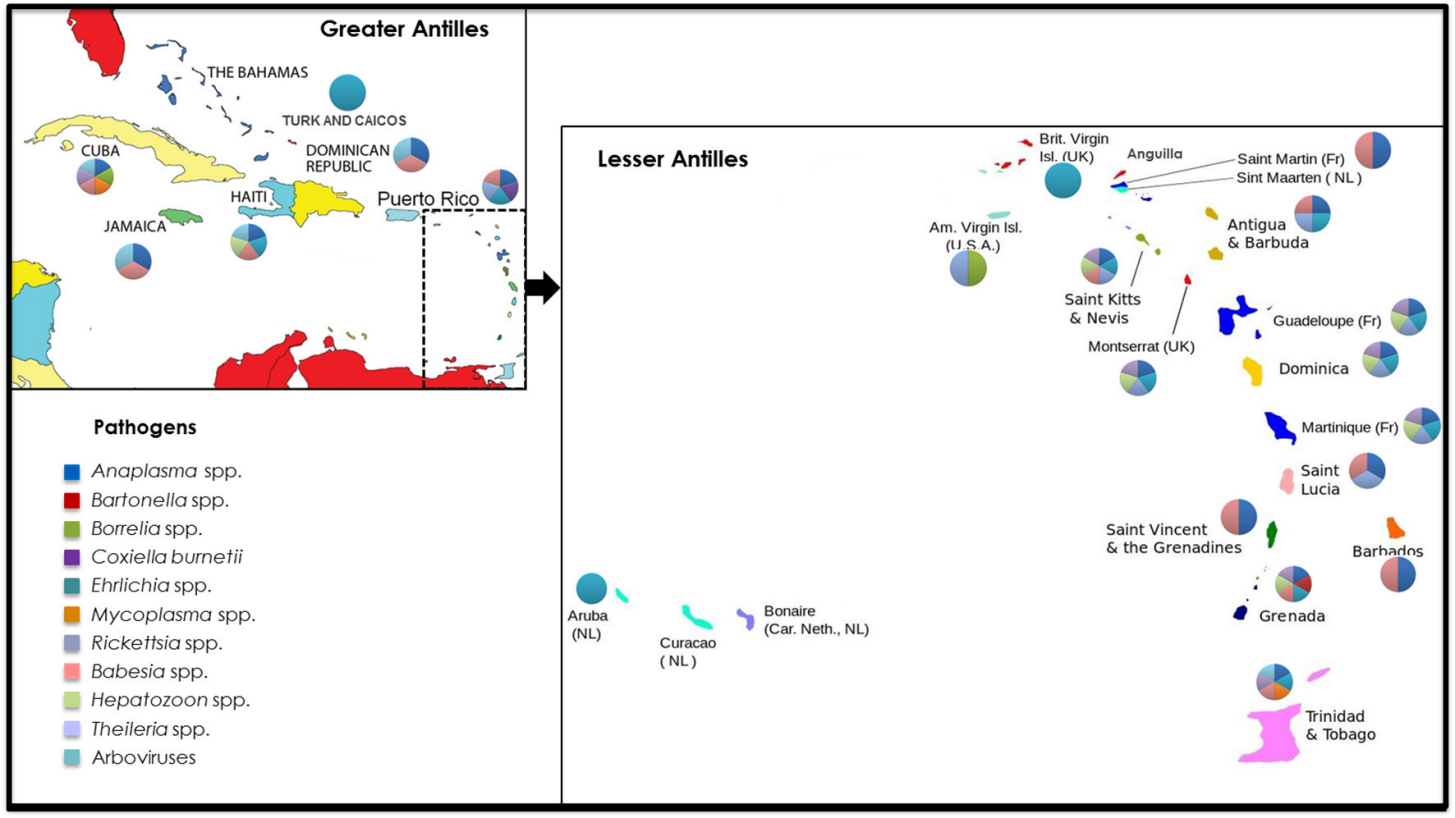
Current and historical distribution of tick-borne pathogens reported in the West Indies. The pathogens covered in this review include bacteria (*Anaplasma* spp., *Ehrlichia* spp., *Rickettsia* spp., *Borrelia* spp., *Bartonella* spp., *Mycoplasma* spp., *Coxiella* spp.), protozoa (*Babesia* spp., *Theileria* spp., and *Hepatozoon* spp.), and arboviruses (African swine fever virus, Soldado virus, Hughes Virus, Wad Medani Virus, and Estero Real Virus). Pathogen groups and political borders are illustrated by different colors in the representative pie charts and key.

TBPs affecting human and animal health in the West Indies include bacteria (*Anaplasma* spp., *Ehrlichia* spp., *Rickettsia* spp., *Borrelia* spp., *Bartonella* spp., *Mycoplasma* spp., *Coxiella* spp.), protozoa (*Babesia* spp., *Theileria* spp., and *Hepatozoon* spp.), and arboviruses (African swine fever virus, Soldado virus, Hughes Virus, Wad Medani Virus, and Estero Real Virus). An overview of the current and historical distribution of these pathogens within the Caribbean region is presented in Figure 1 and Table 1.

### The main economically important TBPs in the Caribbean

Due to their economic impact, six major animal TBPs are actively monitored in the Caribbean: *Anaplasma marginale, Ehrlichia ruminantium, Babesia bovis, Babesia bigemina, Babesia caballi*, and *Theileria equi*.

*Anaplasma marginale, Ehrlichia ruminantium, Babesia bovis*, and *Babesia bigemina* are mainly cattle pathogens that can cause significant economic losses in both dairy and beef industries (Camus and Barre, 1995). TBDs induced by these pathogens are listed by the World Organisation of Animal Health (WOAH) as notifiable diseases^1^. In addition to the direct impact on cattle health, the endemicity of these pathogens is a substantial hindrance to livestock development in the Caribbean. In particular, introducing high-productive breeds into the region is risky due to their increased susceptibility to TBPs compared to the more resistant, but less productive, local creole cattle. Most of the recorded clinical TBD cases have occurred in imported animals, mainly dairy cattle (Camus and Montenegro-James, 1994; Allsopp, 2015).

*Anaplasma marginale*, responsible for bovine anaplasmosis, is mechanically transmitted by biting flies or blood-contaminated fomites, or biologically by ticks, according to the strain (Kocan et al., 2010). Within the Caribbean, *Rh. microplus* ticks are considered as vector of *A. marginale*. It has been experimentally demonstrated that *Rh. microplus* can transstadially and intrastadially transmit this bacterium (Connell and Hall, 1972). However the importance of those phenomenons under natural conditions need to be explored, as *Rh. microplus* are one-host tick, meaning that most of the specimens spend their life on the same host (Connell and Hall, 1972; Alonso et al., 1992; Aguirre et al., 1994; Futse et al., 2003). *Anaplasma marginale* is endemic throughout most of the Caribbean islands, with seroprevalence rates in the Lesser Antilles ranging from 1 to 68% (Camus and Montenegro-James, 1994; Camus and Barre, 1995).

*Babesia bovis* and *Babesia bigemina*, both transmitted by *Rh. microplus*, are the causative agents of bovine babesiosis. *Rh. microplus* adult females acquire haemoparasites during blood meals from an infected host, and then transovarially transmit it to their offspring, leading to infected larvae. Then, two different ways of transmission to a new host are observed according to the parasite species. *Babesia bovis* is transmitted by larvae, whereas B. bigemina is transmitted by nymph and to a lesser extent by adult ticks (Alonso et al., 1992). Both *Babesia bovis* and *Babesia bigemina* parasites are endemic to the Caribbean islands, with seroprevalence rates within the Lesser Antilles ranging from 18 to 58%, and 22 to 69%, respectively (Camus and Montenegro-James, 1994; Camus and Barre, 1995; Urdaz-Rodríguez et al., 2009).

*Ehrlichia ruminantium* (formerly *Rickettsia ruminantium* or *Cowdria ruminantium*) is responsible of bovine erhlichiosis, commonly called cowdriosis or heartwater. Bovine erhlichiosis is fatal to cattle, sheep, goats, and some wild ruminants, and is transmitted by *A. variegatum* in the West Indies (Camus and Barré, 1992). Ticks mainly transmit *E. ruminantium* transstadially, thus larvae or nymphs must acquire infections before they can transmit this bacterium to subsequent nymphal or adult stages (Bezuidenhout, 1987). Transovarial transmission and intrastadial transmission have been reported by female and male *A. hebreum* respectively, however, none of those phenomenons have been recorded in *A. variegatum* (Bezuidenhout and Jacobsz, 1986; Andrew and Norval, 1989; Allsopp, 2015). Heartwater is endemic to the islands of Guadeloupe, Marie-Galante, and Antigua. Between 2003 and 2005, *E. ruminantium* prevalence in *A. variegatum* ticks reached 37% in Guadeloupe, 36% in Marie-Galante, and 6% in Antigua (Vachiéry et al., 2008). Serological evidence for this pathogen was reported in Martinique and Montserrat in asymptomatic sheep, but results suggested potential cross reactions with closely-related *Ehrlichia* spp. (Camus and Barre, 1995; Zhang et al., 2015a). Although, *E. ruminantium* currently seems to be restricted to three islands, the wide distribution of the *A. variegatum* vector throughout the West Indies indicates that the risk of pathogen establishment in current *E. ruminantium*-free areas remains (Kelly et al., 2011; Allsopp, 2015).

Finally, *T. equi* (formerly *Piroplasma equi, Nuttalia equi*, or *Babesia equi*) and *B. caballi* (formerly *Piroplasma caballi*) both induce equine piroplasmosis. This TBD is also listed as notifiable by the WOAH and is heavily monitored during horse importation into free-status countries. The introduction of asymptomatic infected carriers in disease-free areas where competent tick vectors are present, can lead to the spread of piroplasms to susceptible animals, and would lead to significant economic losses for the horse racing industry (Asgarali et al., 2007; Wise et al., 2013). Multiple ixodid ticks are suspected to be vectors of piroplasmosis, such as *Dermacentor, Hyalomma, Rhipicephalus*, and *Amblyomma* spp. Experimental and natural data are however needed to confirm the role of these ticks in equine piroplasmosis epidemiology (Wise et al., 2013). The principal tick species suspected to be responsible for parasite transmission within the Caribbean is the one-host tick *D. nitens*. Generally, *B. caballi* is transstadially and transovarially transmitted in ticks which remain as infection reservoirs. *T. equi* parasites are transstadially or intrasstadially maintained in ticks, such that nymphs and adult ticks can then transmit this pathogen to their hosts (Uilenberg, 2006; Scoles and Ueti, 2013). Even if these two parasites are considered endemic in most countries with tropical and subtropical climates, data concerning their importance and epidemiology within the Caribbean is limited. A previous study in Trinidad reported seroprevalences of 33.3% for *T. equi* and 68.8% for *B. caballi* (Asgarali et al., 2007). Two other studies detected *T. equi* in Nevis, Dominica, and St Kitts, and *B. caballi* in Grenada, Monserrat, and St Kitts, in several ruminants species using molecular tools (Li et al., 2015; Zhang et al., 2015b).

### Other TBPs reported in the Caribbean

Canine monocytotic ehrlichiosis caused by *Erhlichia canis* has been reported within the Carribean and is associated with the tick vector *Rh. sanguineus*. Frequently considered as a canid pathogen, *E. canis* has also been suspected to cause human disease in Venezuela, suggesting a potential zoonosis risk (Perez et al., 2006; Starkey et al., 2016).

*Ehrlichia canis* or other closely related species have been detected by *Ehrlichia* FRET-qPCR and by sequencing portions of the 16SrRNA and gltA genes in ruminant blood samples from four Caribbean islands (Zhang et al., 2015a). The highly conservative nature of the genes targeted for the assay can not allow the identification of the *Ehrlichia* species occurring in those samples. However, those results may suggest that (1) if *E.canis* is present in those samples, thus this bacterium usually infecting dog can be able to infect a broader host range, including ruminants (2) if it is not *E. canis*, thus a new closely related *Ehrlichia* species can be present in the Caribbean, infecting ruminants (Zhang et al., 2015a). In addition, *Erhlichia minasensis (E. mineirensis)*, a new cattle pathogen closely related to *E. canis*, has been detected in *Rh. microplus* ticks, in cattle from the Americas (Canada and Brazil), and from Tahiti, French Polynesia (Aguiar et al., 2014; Cabezas-Cruz et al., 2016; Laroche et al., 2016). Further studies should address whether *E. minasensis* is also present in the Caribbean and if it is responsible for the *Ehrlichia* spp.-positive samples previously described.

In the USA, *Panola Mountain Ehrlichia* sp. (PME) is a recently described human pathogen mainly transmitted by *A. americanum*, and which is circulating in ruminant populations (Reeves et al., 2008). PME, which is closely related to *E. ruminantium*, has been detected in *A. variegatum* collected on livestock from Dominica and St Kitts (Loftis et al., 2016). Further studies on the vector competence of *A. variegatum* in PME transmission are needed, however this study suggests that this pathogen circulates in the livestock and tick populations on those two islands. As *A. variegatum* can feed on humans, PME's zoonotic risk should be evaluated (Loftis et al., 2016).

*Anaplasma platys* is a dog pathogen responsible for canine cyclic thrombocytopenia, commonly transmitted by *Rh. sanguineus* and is reported in Caribbean dog populations (Starkey et al., 2016). Typically considered as a canid pathogen, *A. platys* is also suspected to be responsible for human disease in Venezuela. Blood samples from two patients exposed to *Rh. sanguineus* and presenting nonspecific clinical signs (headaches and muscle pains), tested positive for *A. platys*, and intra-platelet inclusion bodies consistent with this pathogen were also observed (Arraga-Alvarado et al., 2014). These results suggest a potential risk of zoonosis (Arraga-Alvarado et al., 2014; Starkey et al., 2016). The occurrence of tick-transmitted canine pathogens that also appear to be zoonotic, suggests that there are potential sanitary risks associated with stray dog populations, which are largely distributed throughout the Caribbean islands. Stray dogs could therefore be involved in TBP epidemiology, potentially acting as synanthropic infectious reservoirs, creating potential risks for human populations and domestic animals (Qablan et al., 2012). The zoonotic potential of these pathogens highlights the need for increased awareness and communication with medical community.

*Anaplasma phagocytophilum* is a well-characterized human and animal pathogen in the Northern Hemisphere, and induces granulocytic anaplasmosis. This pathogen is usually transmitted by hard ticks of the *Ixodes persulcatus* complex and infects a large range of hosts worldwide. While *A. phagocytophilum* is linked to human granulocytic anaplasmosis in the USA, in Europe it is mainly associated with tick-borne fever in ruminants (Dugat et al., 2015). Detection of antibodies to *A. phagocytophilum* in canine sera was reported in Puerto Rico and Grenada (Yabsley et al., 2008; McCown et al., 2013). However, the low specificity serological assay combined with the lack of knowledge on *Ixodes* spp. distributed in the Caribbean (reported in Puerto Rico but not in Grenada) suggest that those positive results were likely due to non-specific cross-reactions with *A. platys* (Yabsley et al., 2008; McCown et al., 2013).

*Rickettsia africae* belongs to the spotted fever group of the genus *Rickettsia* and is the etiologic agent causing African tick bite fever. In the Caribbean, *R. africae* is transmitted by *A. variegatum* (Parola et al., 1999). Despite high levels of tick infection and a seroprevalence ranging from 7 to 62% in human and animal sera, only one human case of African tick bite fever has been officially reported in a French tourist traveling in Guadeloupe (Parola et al., 1998, 1999; Kelly et al., 2003, 2010a; Robinson et al., 2009).

*Rickettsia conorii*, another spotted fever group pathogen, is the etiological agent of Mediterranean spotted fever and has also been reported in the area, but the actual occurrence of this pathogen is uncertain, as non-specific serological tests were used (Morel, 1967; Parola et al., 1999).

Although some *Rickettsia* are usually transmitted by fleas or lice, ticks are also suspected to be involved in their epidemiology, such as for *Rickettsia felis* and *Rickettsia typhi*, both only detected once in the Carribean (Tamsitt and Valdivieso, 1970; Kelly et al., 2010b).

Lyme disease is a borreliosis caused by a complex of ~18 genospecies of spirochete known collectively as *Borrelia burgdorferi* sensu lato (s.l.), and is the most common vector-borne disease affecting humans in the Northern Hemisphere. The life cycle of these pathogens is especially complex, involves numerous vertebrate hosts, and are only known to be transmitted by *Ixodes* spp. (Schotthoefer and Frost, 2015). Whether *B. burgdorferi* sensu lato spirochetes circulates within the Carribean, where typical vector ticks are absent, is a controversial topic. *Borrelia burgdorferi* sensu stricto antibodies were detected in human serum in Cuba, associated with clinical cases of Lyme disease-like syndrome (Rodríguez et al., 2004, 2012). However, (Dessau, 2012), clearly reject the interpretation of the serological assay used to detect antibodies to spirochetes in the Cuban study, and instead consider that the positive results are more likely serological background noise. Moreover, the observation of erythema migrans-like skin lesions are not pathognomonic for Lyme disease diagnosis, and does not in itself prove the presence of spirochetes (Sharma et al., 2010; Lantos et al., 2013). Further studies, especially those isolating spirochetes, should be performed to elucidate whether *B. burgdorferi* sensu lato spirochetes circulates within the Carribean.

Relapsing fever group *Borrelia* spp. have been reported in the US Virgin Islands, with the detection of antibodies to *Borrelia hermsii*, or a closely related *Borrelia* species, in association with a human case of relapsing fever. Relapsing fever group *Borrelia* spp. are usally transmitted by soft ticks, suggesting the presence of *Ornithodoros* species on the island, the disease case was declared autochtonous (Flanigan et al., 1991). Given the significant medical and economical impact of relapsing fever, further studies are required to identify spirochetes and their potential tick vectors circulating in the West Indies.

*Bartonella vinsonii* subspecies *berkhoffii*, is a recent human and dog pathogen, and is mainly responsible for vascular and neurological infections (Breitschwerdt et al., 2010). This pathogen has been reported in Caribbean dog populations (Yabsley et al., 2008). The way of transmission of this pathogen is not know, but dog's ectoparasites such as tick and fleas are suspected to play a role as vectors. Indeed, the fact that *Bartonella vinsonii* subspecies *berkhoffii* infections in dogs are associated with tick infestations and with other TBPs co-infections suggest that tick may be able to transmit it (Billeter et al., 2008; Yabsley et al., 2008). As *Rh. sanguineus* is the main dog associated tick species occurring in the Caribbean, its vector competence for *Bartonella vinsonii* subspecies *berkhoffii* need to be investigate. Even if (Billeter et al., 2012) demonstrated the experimental failure of transovarial transmission of this bacterium by *Rh. sanguineus*, other ways of tick-assisted transmission remain to be explored (Billeter et al., 2008, 2012; Yabsley et al., 2008).

*Coxiella burnetii* (formerly *Rickettsia burnetii*) is a small obligate intracellular gram-negative bacterium producing Q fever, a human disease occurring worldwide. Q fever is considered to be an airborne zoonotic disease, mainly transmitted through contaminated aerosols such as barnyard dust contaminated by dried placental material, birth fluids, and excreta of infected animals. However ticks such as *Rh. microplus* or *Rh. sanguineus* are also suspected to be involved in Q fever epidemiology as potential vectors (Duron et al., 2015). Little is known about *C. burnetti* distribution within the Caribbean, and only one older reference reported its detection in Puerto Rican cattle (Tamsitt and Valdivieso, 1970).

*Mycoplasma haemocanis* and *Candidatus* M. haematoparvum, responsible for canine mycoplasmosis, have been reported in the Caribbean. Both pathogens are suspected to be transmitted by *Rh. sanguineus* (Barker et al., 2010). Mycoplasmosis is not considered to be a TBD, but some hemotrophic mycoplasms of veterinary and public health importance are occasionally detected in ticks, and in co-infections with known TBPs in affected hosts. Ticks are therefore suspected to play a role in the epidemiology of these bacteria (Barker et al., 2010). Feline hemoplasma, *Mycoplasma haemofelis* and *Candidatus* Mycoplasma haemominutum, have been reported in cats with severe anemia in Trinidad (Georges et al., 2008). In addition, *Mycoplasma wenyonii* and *Mycoplasma ovis*, infecting cattle and sheep respectively, have been reported in Cuba (Rodríguez et al., 1989a,b). However, tick involvement in pathogen epidemiology is unknown.

*Babesia canis vogeli* and *Babesia gibsoni* are transmitted by *Rh. sanguineus*. Both protozoan parasites cause canine babesiosis. Although these parasites are usually associated with dogs, pathogen DNA has been detected in unusual vertebrate hosts such as cats, cattle, goats, sheep, and donkeys in the Caribbean, suggesting a larger host range than previously thought (Georges et al., 2008; Li et al., 2015). Cattle parasitized by *Rh. sanguineus* have not been reported in the Caribbean, suggesting the presence of another vector. *Rhipicephalus turanicus* is a tick species that is difficult to differentiate from *Rh. sanguineus*, and has been put forward as a potential vector for *B. canis vogeli*. No reports of *Rh. turanicus* in the West Indies exist, but since *Rh. turanicus* can parasitize a broader range of hosts, including cattle, its potential presence in the area needs further investigation (Li et al., 2015).

*Babesia canis rossi* and *Babesia vulpes* are other canid pathogens that have also been detected in small ruminants in Montserrat and Dominica (Li et al., 2015; Zhang et al., 2015b). Usually, these two pathogens circulate in the Northern Hemisphere in parallel with the *Haemaphysalis* spp. tick vector. The detection of *B. canis rossi* and *B. vulpes* in small ruminants where there is no record of known tick vector, deserves further epidemiological investigation to understand their potential circulation in the West Indies (Li et al., 2015).

Bovine theileriosis has rarely been reported in the Caribbean, and infections are typically asymptomatic in domestic and wild ruminants. *Theileria mutans* and *Theileria velifera* have been reported in Caribbean cattle, and are transmitted by *A. variegatum* (Uilenberg et al., 1983; Alonso et al., 1992). One report described *Theileria parva* infection in Guadeloupe, but the study suggested that *T. mutans* was misidentified (Morel, 1967). New species of unknown pathogenicity have been recently detected, including *Theileria* sp. NG-2013a, *Theileria* sp. OT3, *Theileria* sp. YW-2014, and *Theileria* sp. B15a, in various domesticated Carribean animals (Zhang et al., 2015a). Further studies are needed to identify potential tick vectors and hosts and to determine their potential pathogenicity (Zhang et al., 2015b).

*Hepatozoon canis* is a haemoprotozoan parasite infecting dogs, and is usually transmitted by ingesting infected *Rh. sanguineus* ticks (Starkey et al., 2016)*. Hepatozoon canis* have been reported in dogs from Aruba, Grenada, Haiti, St Kitts, and Trinidad (Sant et al., 2017).

African swine fever virus (ASFV) causes severe haemorrhagic disease in domestic pigs, and can be transmitted by direct contact or by *Ornithodoros* tick bites. ASFV outbreaks have occurred in Cuba, Haiti, and the Dominican Republic in the 1970s, where *Ornithodoros* species, such as *O. coriaceus, O. parkeri, O. turicata*, and *O. puertoricensis*, were suspected as vectors (Butler and Gibbs, 1984; Penrith, 2009). However, ASFV has been since eradicated from the region.

Several arboviruses of unknown pathogenicity have been described in the Carribean region. Arboviruses described within the West Indies are mainly associated with seabird colonies and their ticks (Table 1). Birds are implicated in tick dispersion, suggesting potential viral dissemination risks, and highlighting the need for further epidemiological and pathogenicity studies (Labuda and Nuttall, 2004). As the majority of studies surveying TBPs worldwide have focused on bacterial and/or protozoal pathogens, tick-borne virus diversity is poorly understood (Labuda and Nuttall, 2004; Moutailler et al., 2016b).

## New insights for tick-borne disease epidemiology and control in the West Indies

### Challenges of TBD management in the Caribbean

The management and prevention of TBDs are included within the “*One Health”* global health strategy, although they are still overlooked by other major zoonotic diseases such as rabies, avian influenza, etc… (Dantas-Torres et al., 2012; Gebreyes et al., 2014). The “*One Health”* concept describes a comprehensive health crisis management and prevention approach that encompasses human, animal, and ecosystem health (Day, 2011; Gebreyes et al., 2014). Applying the “*One Health”* approach to TBDs mainly relies on the implementation of enhanced cross-sector communication and the development of technical expertise (Dantas-Torres et al., 2012; Baneth, 2014). In addition to the emergence of animal and zoonotic infectious diseases in the Caribbean and their resulting significant economic impact, many of these islands must cope with limited resources and few available experts, thus preventing standardized and coordinated TBD management and control^2^. However, health networks^3^ have recently been developed in the Caribbean, as an effort to improve the situation. CaribVET^4^ is one of the most developed networks, dealing with animal health and bringing together 45 partners, including the veterinary services of 34 Caribbean and American countries/territories, and international research and health organizations such as UWI Jamaica-Barbados-Trinidad and Tobago, CENSA Cuba, CIRAD Guadeloupe, FAO, and PAHO. CaribVET recently launched a “*One Health”* project to identify a wide range of TBPs affecting both humans and animals across eight Caribbean islands. The results of this study will certainly contribute valuable new information about the current distribution of Caribbean TBDs, and help decision-makers with their risk evaluation and management. Even if such initiatives are carried out and improve overall Caribbean health, progress will always be hampered by the highly contrasting circumstances throughout the region. Such situations include heterogeneous countries and territories defined by differents cultures, demographics, politics, socio-economics, landscapes, and various animal production systems and disease priorities that do not facilitate the implementation of a “*One Health”* TBD management strategy^2^. Indeed, technical, administrative, and socio-economic weaknesses have contributed to the delay and have thwarted large tick eradication programs against *A. variegatum* and *Rh. microplus* within the Caribbean (Crom, 1992; Pegram et al., 2000, 2007). In addition, methods commonly used for tick control are generally based on intensive chemical approaches. The use of acaricides requires frequent and regular treatments which are costly, and contamination of meat, milk, and the environment with chemical residues, and acaricide resistance is a reality. Thus the development of anti-tick vaccines is a promising alternative for tick control (Rodríguez-Mallon, 2016). Since the 90s, anti-tick vaccines have been developed targeting the Bm86 glycoprotein of *Rh. microplus*. The GAVAC and TickGARD vaccines were developed in Cuba and Australia, respectively (De la Fuente et al., 2007). In Cuba, field trials demonstrated that GAVAC vaccination associated with integrated tick control strategies can reduce *Rhipicephalus* spp. infestation, the frequency of required acaricide treatments, and the incidence of babesiosis and anaplasmosis (Valle et al., 2004; De la Fuente et al., 2007).

Thus, to develop efficient tick control programs and prevention strategies in the Caribbean, the following crucial barriers must be overcome: (1) improving epidemiological knowledge on interactions between tick species, circulating pathogens, associated vertebrate hosts including humans and animals, and their environment; (2) development of effective detection and diagnostic tools vital to improving TBD surveillance capacity; and (3) improvement of global communication between all involved sectors, including researchers, physicians, veterinarians, and governmental services, from local to international levels (Dantas-Torres et al., 2012). Successful TBD management will depend on a unified and standardized vision of Carribean health which necessarily relies on existing health networks such as the CaribVET and the Caribbean Vector-Borne Diseases Network (CariVecNet), with technical and financial support from international bodies.

### Tick and TBP epidemiology in the next-generation sequencing era

The extent of tick infestations and the pathogens they can transmit to humans and animals in the Caribbean region is poorly characterized due to a clear lack of actual epidemiological data.

Most of the TBPs reported here have been detected by standard serological or molecular tools (Table 1). However, the majority of the serological TBP assays available in the Caribbean are archaic with likely non-specific cross-reactions, leading to potential pathogen mis-identification and incorrect epidemiological conclusions. For example, the detection of serum antibodies to *A. phagocytophilum* (a zoonotic pathogen responsible for granulocytic anaplasmosis) in Puerto Rican dogs was likely due to non-specific cross-reaction with *A. platys* (Yabsley et al., 2008; McCown et al., 2013). Similar conclusions have been applied to records of serological detection of *R. conorii* in Guadeloupe, which was likely confused with non-specific *R. africae* cross-reactions (Morel, 1967; Parola et al., 1999). Advances in molecular biology since the late 80s, including the use of conventional PCR or real-time PCR, have overcome the specificity and sensitivity limits of serological assays. Moreover, these technologies enable the detection of pathogens in vector ticks. As a result of this technology, the list of potential TBPs is rapidly increasing worldwide, where new potential tick vectors and host associations are continually being described (Estrada-Peña et al., 2013). For example, PCR assays enabled the first detection of PME in *A. variegatum* ticks in St Kitts and Dominica, suggesting that the range of tick vectors can be extended further than previously thought (Loftis et al., 2016). Additionally, the recently-developed generic oligonucleotide FRET-qPCR enables the detection of unexpected pathogen species in cattle, such as *B. gibsoni* and *B. canis vogeli*, and the detection of uncharacterized species, such as *Ehrlichia* spp., which is closely related to *E. canis* bacteria (Li et al., 2015; Zhang et al., 2015a,b). However, pathogen surveillance using these approaches is still limited to the detection of a small set of given pathogens (Estrada-Peña et al., 2013).

Innovative next-generation sequencing (NGS) techniques, combined with bioinformatic analyses enables the construction of a wide and without *a priori* inventory of tick-borne microorganisms, boosting the epidemiology and diagnosis of infectious diseases such as TBDs (Carpi et al., 2011). Several second-generation sequencing (SGS) methodologies are now available (Mardis, 2008; Liu et al., 2012), and some have been used to explore the complexity of bacterial communities within ticks. 16S rRNA tag-encoded amplicon pyrosequencing, Illumina, or 454 pyrosequençing have also been used to describe the structure and diversity of bacterial communities in *Rh. microplus* and *I. ricinus* ticks (Andreotti et al., 2011; Carpi et al., 2011). The main bacterial taxa were characterized, but due to the sequencing approach used, microorganisms were not as easily identified at the species level. The choice of methodology used must relate to the epidemiological purpose of the study, especially if distinction between endosymbiotic and pathogenic bacteria belonging to the same genera is required (Carpi et al., 2011). In addition to pathogen surveillance, NGS technologies now provide opportunities to characterize the tick microbiome. Microbiomes include all the commensal, symbiotic, and pathogenic microorganisms that interact both with each other and the tick, and which likely have a strong impact on tick biology and pathogen transmission ability (reviewed by Narasimhan and Fikrig 2015). Deciphering tick microbiome complexity and function could be a key stepping-stone in the development of new TBD control strategies (Narasimhan and Fikrig, 2015). In addition to genomics, RNA high-throughput sequencing technologies have also been used to characterize the *I. ricinus* transcriptome, facilitating the identification of replicative tick-borne microorganisms as well as the simultaneous analysis of bacteria, parasites, and viruses (Vayssier-Taussat et al., 2013; Bonnet et al., 2014; Moutailler et al., 2016b). These analyses demonstrate the capacity of RNAseq technology to characterize the vast diversity of the tick microbiome, and to detect known but also new and unsuspected pathogens (Vayssier-Taussat et al., 2013, 2015; Bonnet et al., 2014; Moutailler et al., 2016b). Despite the high-throughput capacity of NGS, a large portion of the generated data is unable to be analyzed, and the frequently short read length sometimes hinders *de novo* genome assembly and sequence analysis, especially in relation to species level microorganism identification. Morevover, NGS data is based on conventional homology-based sequence searches, and thus requires specific references. Current genomic reference databases are lacking in accuracy and richness, leading to a significant number of unassigned sequences. As this “black box” may include sequences of new or poorly characterized microorganisms, bioinformatic tools need to be developed in order to overcome such limitations. For example, the recently-developed Batch Learning Self-organizing Maps (BLSOMs) approach has enabled the characterization of bacterial communities from several tick species without the need for taxonomic reference information (Nakao et al., 2013).

Third-generation sequencing (TGS) technologies are now available. Contrary to the sequencing methods described above, TGS technologies, such as Pacbio and MinION, directly target single DNA molecules, enabling real-time sequencing, with long reads ready for analysis immediately after sequencing (Rhoads and Au, 2015; Lu et al., 2016). These innovative technologies offer new opportunities in pathogen surveillance and clinical diagnostic applications. Moreover, the MinION device, with its small size and low cost, can be easily transported into the field during disease outbreaks, greatly aiding real-time pathogen monitoring (Lu et al., 2016). For example, MinION technology was used to sequence Zika virus from clinical samples during the 2016 South-American Zika outbreak (Faria et al., 2017; Quick et al., 2017). Table 2 compares the general features and applications of the main sequencing platforms (the three generations).Finally, the recent development of such high-throughput detection technologies now provides an unprecedented level of rapid and simultaneous tracking of a wide range of pathogens of sanitary importance in tick samples. Combined with the high-throughput sequencing technologies described above, these powerful investigation methods represent a major improvement in TBP and TBD epidemiology and health surveillance. Recently, a high-throughput real-time microfluidic PCR was developed for large and rapid screening of TBPs within European ticks. This detection tool can simultaneously monitor the circulation of 25 bacterial and 12 protozoan species within 94 tick samples (Michelet et al., 2014). Beside the high detection yield, this method is also time and cost saving. Two size of support for microfluidic PCRs are available, a 96.96 chip and a 48.48 chip, which perform in one run 9216 and 2304 real-time PCR reactions respectively. Experiment leading with the 96.96 dynamic array (48.48), take around 4 h (three hours), and the cost per reaction is around $9.1 ($5.5) from tick homogenates to real-time PCR results (Michelet et al., 2014). Such analyses not only provide a rapid overview of pathogen prevalences in field tick samples, but are also able to detect co-infection. Pathogen co-infections, or symbiotic-pathogen associations, play a crucial role in TBD epidemiology. Symbiotic bacteria can affect the vector competence of the tick, and pathogen co-infection can enhance disease severity as well as affecting diagnosis and treatment capacity (Diuk-Wasser et al., 2016; Moutailler et al., 2016a). High-throughput technologies thus represent a major improvement in surveillance methods, suitable for large-scale epidemiological studies, and which can be easily adapted for the monitoring of emerging pathogens from different areas of the world, such as the Caribbean. However, the increased performance of these methods should not conceal the increased risk of epidemiological mis-interpretation of data on pathogen transmission by ticks. Indeed, DNA or RNA detection alone does not conclusively establish that ticks can transmit pathogens and thus cause disease. Ticks harvested directly from the host may have only sampled pathogens present in the host while they themselves remain resistant to the infection and are unable to transmit these pathogens to other susceptible hosts (Loftis et al., 2016). Thus, the physiologic state of the tick (flat, partially fed, mated, engorged, etc…) should be clarified as it can influence the epidemiological meaning of TBPs detection studies (Loftis et al., 2016). Finally, vector competence and the capacity of ticks to transmit an infectious agent, as well as natural evidence of infection are thus needed to prove tick involvement in a pathogen's life cycle (Dantas-Torres et al., 2012).

**Table 2:** Performance comparison of sequencing platforms of the first, second and third generation (G) of sequençing.

**F**	**G**	**Instrument**	**Sequençing technology**	**Maximum read length**	**Accuracy**	**Maximum Reads/Run**	**Time/Run**	**Intrument/and run price ($)**	**Application**
1	1	Sanger, 3730xl DNA Analyzer	Sanger, Chain termination sequençing	1,000b	>99.999	96 (capillaries)	20–180 min	ND	Targeted amplicon sequençing; Bacterial, fongal, viral identification; Microsatellite and STR analysis
	2	Ion Torrent Proton PII	Sequencing by synthesis	100b	>99%	330 M	2–4 h	1,419 K/1 K	Large (human, plant, animal) (Proton PII) and Small (microbe, virus) whole-Genome Sequencing; Metagenomic; Transcriptomic; ChIP- Seq; Exome; Targeted DNA and RNA Sequencing
		Ion Torrent Proton PI		200b	>99%	82 M	2–4 h	149 K/1 K	
		Ion Torrent PGM 318^a^		400b	>99%	5.5 M	4–7 h	50 K/349	Small Whole-Genome Sequencing (microbe, virus); Targeted DNA and RNA Sequencing;ChIP- Seq
		Ion Torrent PGM 316^a^		400b	>99%	3 M	3–5 h	50 K/349	
		Ion Torrent PGM 314^a^		400b	>99%	0.6 M	2–4 h	50 K/349	
		SOLiD 5500xl	Sequençing by ligation	2 × 60b	99.99%	800 M	6 days	595 K/10 K	Human Whole Genome; Small Whole-Genome Sequencing (microbe, virus); Metagenomic; Transcriptomic; ChIP- Seq; Exome; Targeted DNA and RNA Sequencing
2		Genome Sequencer FLX+	Pyrosequencing	1,000b	>99%	1 M	23 h	500 K/6 K	Small Whole-Genome Sequencing (microbe, virus); Targeted Gene and RNA Sequencing; Transcriptomic (FLX+); Metagenomic (FLX+)
		Genome Sequencer Junior^a^		700b	>99%	0.1 M	10 h	125 K/1 K	
3		HiSeq 2000	Sequencing by synthesis	2 × 100b	99.9%	2B	8 days	ND	Large (human, plant, animal) and Small (microbe, virus) whole-Genome Sequencing; Targeted DNA and RNA Sequencing; Exome Sequencing; Transcriptomic; Epigenetic; Shotgun Metagenomic
		HiSeq 2500 High-Output Run/Rapid-Run mode		2 × 125 – 2 × 250b	99.9%	4B−1.2B	6 days−60 h	740 K/29 K	
		HiSeq 3000		2 × 150b	99.9%	2.5B	<1–3.5 days	ND	
		HiSeq 4000		2 × 150b	99.9%	5B	<1–3.5 days	ND	
		NextSeq 500		2 × 150b	99.9%	400 M	12–30 h	250 K/4 K	
		Miseq^a^		2 × 300b	99.9%	25 M	4–55 h	125 K/1.4 K	Small Whole-Genome Sequencing (microbe, virus); Targeted DNA and RNA Sequencing; Epigenetic; 16S Metagenomic (MiSeq)
		Miniseq^a^		2 × 150b	99.9%	25 M	4–24 h	ND	
4	3	PacBio RS II: P6-C4	SMRT (single molecule real-time) sequencing	>20 kb^b^	>86% – >99.9%^c^	~55 k	0.5–4 h	700 K/400	Large (human, plant, animal) (PacBio Sequel) and Small (microbe, virus) whole-Genome Sequencing; Targeted DNA and RNA Sequencing
		PacBio Sequel		>20 kb^b^	>86% – >99.9%^c^	~365 K	0.5–10 h	350 K/850	
5		Oxford Nanopore MinION	Nanopore sequencing	Variable (50 kb − up to hundreds of kb)	>92% −>97% ^c^	4.4 M^d^	1 min – 48 h	1,000/500–900	Bacterial, fongal, viral identification; Genomic, transcriptomic, epigenetic

Informations obtained from Liu et al. (2012), Rhoads and Au (2015), and Lu et al. (2016); and from the different furnisher (F) web site: (1) Thermo Fisher Scientific, Sanger/Ion Torrent™/SOLiD™: www.thermofisher.com; (2) Roche (454 Life science): http://allseq.com/knowledge-bank/sequencing-platforms/454-roche/; (3) Illumina: https://www.illumina.com/systems/sequencing-platforms.html; (4) Pacific Biosciences: http://www.pacb.com/products-and-services/pacbio-systems/; (5) Oxford Nanopore Technologies: https://nanoporetech.com/index.php/products#comparison

a Desktop advice;

b 20 Kb for half of the data; up to 60 kb;

c consensus accuracy;

d *Number of reads at 10 kb at standard speed*.

### Molecular biology technologies for tick taxonomy

Accurately identifying the tick vector is essential in TBD epidemiological studies, however, tick taxonomy usually relies on morphological specimen identification. Precisely identifying morphological characteristics in ticks can be challenging, and requires expertise in acarology. Moreover, adequate morphological identification keys for all the tick species occurring in a given area, and more particularly for each developmental stage of a tick species, are not always available in the literature (Estrada-Peña et al., 2013). In addition, during tick collection and storage in the field, samples can be damaged or limited to developmental stages such as larvae or nymphs, which are more difficult to identify than adults. Ticks collected in (sub)-tropical areas are often directly sampled from the host because trapping in the environment is cumbersome and at times unsuccessful. Those specimens collected from hosts are often engorged and deformed, thus making morphological determination more difficult (Zhang and Zhang, 2014). Under such circumstances, molecular tools can be useful in speciating ticks. DNA barcoding approaches have been successfully tested for tick molecular identification using reference databases. Owing to the consistency between morphological and molecular assignation for the majority of ticks, DNA barcoding is eminently suitable for taxonomy (Zhang and Zhang, 2014). MALDI-TOF mass spectrometry for tick identification could also become a promising alternative tool as it only requires a single tick leg for the process (Yssouf et al., 2015). Thefore, the combined use of both morphological and molecular tick identification methods together is required to fully decipher the complexity of tick taxonomy. Finally, in regard to tick identification, the characterisation of the diversity of hosts involved in the bio-ecology of ticks through the analysis of the blood meal present in the tick midgut may provide further information on TBPs epidemiology. Briefly, such analyses consist in the detection of fragment of currently known hosts's conserved genes in tick by molecular assays followed by sequence analysis (Collini et al., 2015). Identifying the diversity and the nature of the hosts used for the tick feeding may help to understand which animals are involved in tick life cycle and which can act as reservoir of pathogens (Estrada-Peña et al., 2013; Collini et al., 2015).

## Conclusions

We have reviewed the currently available epidemiological literature on ticks and TBPs circulating in the Caribbean. The majority of reported and monitored TBPs in the Caribbean are those concerning pets and livestock, however, many records are outdated and are based on underperforming serological assays. This situation highlights the need to update epidemiological data concerning the diversity of TBPs circulating in the West Indies. Surprisingly, despite the widespread circulation of zoonotic TBPs, few human TBDs have been reported within the area. Most tick infestation reports in the West Indies have been associated with cattle and dogs. However, as the diagnosis of TBDs is challenging and requires a certain level of medical expertise and awareness, human infections might be under-reported in this region. In addition, several zoonotic TBPs infecting dogs represent potential zoonotic risks that should be addressed. The use of innovative technologies such as high-throughput pathogen sequencing and detection has opened unparalleled capacities to unravel the diversity of TBPs circulating in the area. In particular, high-throughput real-time microfluidic PCR is a powerful tool for the simultaneous detection of up to 96 different pathogens in up to 96 samples, using nano volumes of samples. Additional advantages of this nanotechnology are the reduced level of expertise and shorter time required for sample preparation and data analysis compared to high-throughput sequencing. The application of such technologies for pathogen detection in large-scale surveys is very likely to generate important contributions toward the epidemiology, prevention, and control of TBDs in the Caribbean.

## Author contributions

MG wrote the paper. AC-C, RC, MV-T, EA, and SM reviewed the manuscript. EA and SM supervised the manuscript.

### Conflict of interest statement

The authors declare that the research was conducted in the absence of any commercial or financial relationships that could be construed as a potential conflict of interest. The reviewer JM and handling Editor declared their shared affiliation.

## Footnotes

^1^http://www.oie.int/en/animal-health-in-the-world/oie-listed-diseases-2017

^2^https://www.onehealthcaribbean.org/index.php/aboutus/one-health-one-caribbean-one-love-project

^3^http://www.who.int/tdr/news/2016/VBD_networks_collab_centres.pdf

^4^https://www.caribvet.net/
